# Sphingomyelin Metabolism Modifies Luminal A Breast Cancer Cell Line under a High Dose of Vitamin C

**DOI:** 10.3390/ijms242417263

**Published:** 2023-12-08

**Authors:** Michela Codini, Federico Fiorani, Martina Mandarano, Samuela Cataldi, Cataldo Arcuri, Alessandra Mirarchi, Maria Rachele Ceccarini, Tommaso Beccari, Toshihide Kobayashi, Nario Tomishige, Angelo Sidoni, Elisabetta Albi

**Affiliations:** 1 Department of Pharmaceutical Sciences, University of Perugia, 06126 Perugia, Italy; federico.fiorani@studenti.unipg.it (F.F.); samuela.cataldi@unipg.it (S.C.); mariarachele.ceccarini@unipg.it (M.R.C.); tommaso.beccari@unipg.it (T.B.); 2 Section of Anatomic Pathology and Histology, Department of Medicine and Surgery, University of Perugia, 06126 Perugia, Italy; martina.mandarano@unipg.it (M.M.); angelo.sidoni@unipg.it (A.S.); 3 Section of Anatomy, Department of Medicine and Surgery, University of Perugia, 06126 Perugia, Italy; cataldo.arcuri@unipg.it (C.A.); alessandra.mirarchi@unipg.it (A.M.); 4 UMR 7021 CNRS, Faculté de Pharmacie, Universitè de Strasbourg, 67401 Illkirch, France; toshihide.kobayashi@unistra.fr (T.K.); nario.tomishige@unistra.fr (N.T.); 5 Cellular Informatics Laboratory, RIKEN, Wako 351-0198, Saitama, Japan

**Keywords:** vitamin C, breast cancer, MCF7 cells, MB-231 cells, estrogen receptor, progesterone receptor, HER-2, Ki-67, sphingomyelin, ceramide

## Abstract

The role of sphingomyelin metabolism and vitamin C in cancer has been widely described with conflicting results ranging from a total absence of effect to possible preventive and/or protective effects. The aim of this study was to establish the possible involvement of sphingomyelin metabolism in the changes induced by vitamin C in breast cancer cells. The MCF7 cell line reproducing luminal A breast cancer and the MDA-MB-231 cell line reproducing triple-negative breast cancer were used. Cell phenotype was tested by estrogen receptor, progesterone receptor, human epidermal growth factor receptor 2 expression, and proliferation index percentage. Sphingomyelin was localized by an EGFP-NT-Lys fluorescent probe. Sphingomyelin metabolism was analyzed by RT-PCR, Western blotting and UFLC-MS/MS. The results showed that a high dose of vitamin C produced reduced cell viability, modulated cell cycle related genes, and changed the cell phenotype with estrogen receptor downregulation in MCF7 cell. In these cells, the catabolism of sphingomyelin was promoted with a large increase in ceramide content. No changes in viability and molecular expression were observed in MB231 cells. In conclusion, a high dose of vitamin C induces changes in the luminal A cell line involving sphingomyelin metabolism.

## 1. Introduction

Vitamin C (VitC) or ascorbic acid is a molecule synthesized by all plants and the vast majority of vertebrates in which l-gulonolactone oxidase catalyzes the final stage of its biosynthesis [1]. Humans and other primates cannot synthesize VitC due to the mutation of the gene coding for this enzyme [2]. Intestinal absorption, entry into the cells of various tissues, and renal reabsorption of VitC depend on the expression of proteins belonging to the sodium-dependent VitC transporter (SVCT) family [3]. Thus, the different partitioning of VitC in numerous organs and systems is due to the expression of SVCT and its affinity for the substrate [4]. Polymorphisms of the genes responsible for the synthesis of the SVCT protein and their possible variations in activity are responsible for differences in plasma levels and/or possible permanent VitC deficiencies between individuals [5]. Physiological plasma concentrations are obtained by oral ingestion and pharmacological (millesimal) concentrations by parenteral administration [6]. VitC acts on cell metabolism as an electron donor, playing an antioxidant role with a consequent protective role on proteins, fats, and DNA structure/function [6]. VitC is oxidized to an ascorbyl radical and subsequently to dehydroascorbic acid, which has a half-life of a few minutes, and is then converted back to VitC [7]. This recycling process may be impaired in smokers or during diseases such as diabetes, atherosclerosis, heart disease, stroke, macular degeneration [8,9,10,11].

The idea that VitC may be useful in the case of cancer originated in the 1970s [12]. Since then, numerous studies have been conducted with conflicting results, ranging from the complete absence of VitC’s effect in cancer, to its possible protective effect, to its potential therapeutic effect when administered in high concentrations [13,14,15]. Ngo et al. have described cancer vulnerabilities for high-dose vitamin C therapy [16]. Other authors noted that in regard to vitamin C, much of the controversy arose from the lack of predictive biomarkers for stratification of patients, as well as a clear understanding of the mechanism of action and its multiple targets underlying the anticancer effect [17]. Critical differences in the effect on cancer cells between oral and intravenous VitC administration routes have been reported, indicating that millimolar concentrations of VitC can be achieved only when administered intravenously [18]. In a phase I clinical study on patients with an advanced solid tumor or hematological malignancy refractory to standard therapy, 10 mM of VitC was sufficient to slow the growth of cancer cells [19]. The vast majority of studies conducted in humans with the intravenous injection of high doses of vitamin C (50 g and 100 g/day) were carried out on patients in the advanced stages of the disease. The results showed an improvement in symptoms and an extension of life in a percentage of cases ranging from 10 to 22% in relation to the various studies [20]. The variability among studies might be due to the possible different levels in VitC concentration in patients before the treatment, considering that cancer patients could have hypovitaminosis C mainly due to the enhanced oxidative stress and inflammatory processes that increase utilization of ascorbate [21]. In vitro studies showed that VitC at concentrations of 0.25–2.0 mM significantly induced apoptosis in acute myeloid leukemia cell lines [22].

The first observations of the effect of VitC on breast cancer date back over 40 years. In the 1980s, it was shown that long-term administration of 3 g VitC per day did not influence the prognosis of early stage breast cancer [23]. In 2014, a meta-analysis study showed that VitC supplementation was associated with a reduced risk of breast cancer-specific mortality [24]. Over the years, numerous studies have shown that VitC slows the growth of breast cancer cells [25]. 

PR It is clear from the literature that the results on the effect of VitC on breast cancer are different in several studies. The use of different concentrations of VitC could be an explanation. Furthermore, it is important to consider the heterogeneity of the disease both for the diversity of the proliferative index (Ki67) of the different subtypes and for the presence/absence of the expression of estrogen receptors (ER), progesterone receptor (PR), and the human epidermal growth factor 2 (HER2).

Gene expression profiling allowed for the identification of molecular subtypes, namely luminal A (LumA), luminal B (LumB), non-LumA and B, and triple-negative basal-like (TNBC). LumA is ER+ and/or PR+ and HER2−; LumB is further classified into LumB HER2− (ER+ and/or PR+ and HER2−) and LumB HER2+ (ER+ and/or PR+ and HER2+). Non LumA and B tumors are hormone-negative; TNBC is ER−, PR− and HER2− and has the worst prognosis of all subgroups [19]. In addition, a low-claudin (ER−, PR− and HER2−) triple-negative subtype has been described. Using genomic and transcriptomic analyses, 10 breast cancer subtypes were identified based on integrated clusters (4 ER-negative, 6 triple-negative) [26]. A two-sample Mendelian randomization (MR) study aimed at analyzing the role of antioxidants, including VitC, in breast cancer subcategories such as ER+ breast cancer versus ER- breast cancer, showed that VitC has no effect on the risk of developing the disease [27]. Few studies have been conducted in vitro on the effect of VitC on experimental models mimicking breast cancer subtypes. The most studied cell lines are: MCF7 as a LumA model (ER+, PR+/−, HER2−) and MDA-MB-231 as triple-negative claudin-low (ER−, PR−, HER2−) [28]. Subramani et al., 2014 showed that VitC supplementation in MCF7 cells reduced the cytotoxic effect of tamoxifen, indicating that the vitamin could reduce the therapeutic effect of the drug [29]. In the same cell type, VitC appears to enhance the antiproliferative effect of doxorubicin [30]. Ibrahim et al., 2022 demonstrated that VitC concentrations above 82 μM induced a drastic reduction in the viability of MCF7 cells [31]. Khazaei et al., 2022 demonstrated that VitC confers different effects on root viability depending on its concentration and cell type, MCF7 and MDA-MB-231. Pharmacological concentrations of VitC were required after irradiation to reduce the viability of MCF7 cells and before irradiation to reduce the viability of MDA-MB-231 cells [32]. Recently attention has focused on the role of sphingolipids (Sphs) in breast cancer, both for their correlation with cell growth and as possible diagnostic and prognostic markers [33]. The key molecule in Sph metabolism is sphingomyelin (SM), which can be catabolized or resynthesized from Ceramide (CER) by sphingomyelinase (SMase) or SM-synthase, respectively [34]. CER is phosphorylated to CER-1-phosphate by CER kinase (CERK) or is degraded by ceramidase (CERase) to sphingosine (S), which can be converted to sphingosine-1-phosphate (S1P) by sphingosine kinase (SPHK). In addition, CER can be used to synthesize glucosylCER (GCER) or galactosylCER (GalCER). CER synthesis and turnover are altered in breast cancer [35]. Kar et al., 2023 showed a difference between luminal and triple-negative subtypes by identifying only 5 differential Sphs [36]. Notably, the luminal types were characterized by a lower level of CER, GCER and S1P and a higher level of C1P and S compared to the triple-negative types. CERK was altered in triple-negative and triple-positive breast cancer subtypes [37]. Interestingly, CERK has been identified as a regulator of tamoxifen resistance in ER+ breast cancer [38]. A correlation between CERK and SPHK1 with metastasis and/or drug resistance in breast cancer has been demonstrated [39]. The possible mechanism of action of VitC in MCF7 and MDA-MB-231 cells via membrane lipids has been suggested. VitC alone or in combination with somatostatin has been shown to decrease omega-6 linoleic acid and increase omega-3 fatty acids in MCF7 cells and to decrease omega-6 linoleic acid, arachidonic acid, and omega-3 in MDA-MB-231 [40]. It has been claimed that VitC increases the production of sphingoid bases in keratinocytes [41], but there are no data on the effect of VitC on Sph metabolism in breast cancer.

The present study tested the effects of a low, pharmacological concentration of VitC on cell viability and hormone receptor expression in MCF7 and MDA-MB-231 cells in order to understand whether its effect is limited to the reduction of cell growth or whether it can influence the outcome of therapeutic treatment. In addition, the metabolism of SM was studied as a possible signaling mechanism.

The results clearly show that pharmacological concentrations of VitC reduce the viability of MCF7 cells, but also reduce ER expression and induce HER-2 expression. The results suggest that high concentrations of VitC reduce the viability of MCF7 cells and a high dose of VitC, while limiting the growth of LumA breast cancer, makes it less responsive to hormone therapy. No effect of VitC was found in MDA-MB-231 cells. Analysis of Sph metabolism shows a specific enrichment in the Cer content in MCF7 cells.

## 2. Results

### 2.1. Effect of Vitamin C on MCF7 and MB-231 Cell Phenotypes

**Figure 1:**
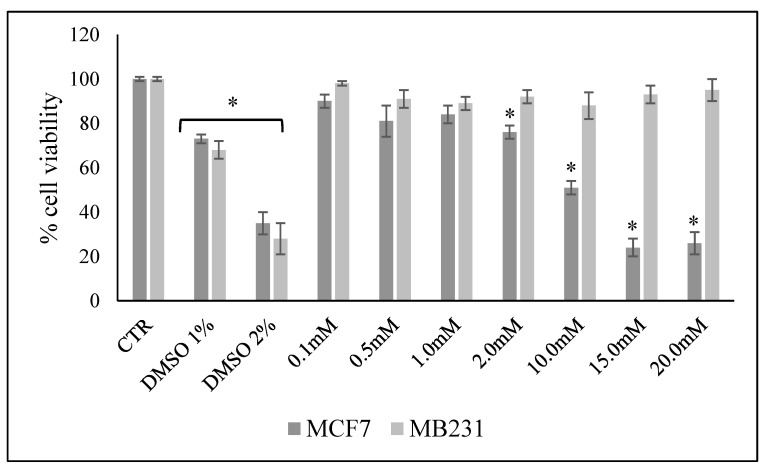
Effect of increasing doses of VitC on MCF7 and MB231 cell viability. Data are expressed as percentages with respect to the control sample (CTR) (100%) and represent the mean ± SD of 3 independent experiments performed in duplicate. DMSO treatment was used as a positive CTR. * *p* < 0.05 versus CTR.

A general overview of the MCF7 and MB-231 cell lines has recently been reported [26]. Here, we attempted to test the possible effect on cell viability of increasing doses of VitC in MCF7 and MB231 cells with the MTT assay after 48 h of cell culture. The results showed no significant differences in the cell viability of MCF7 cells cultured in the presence of 0.1, 0.5 and 1.0 mM VitC compared to control cells (Figure 1). Cell viability was drastically reduced by increasing the VitC dose to 20 mM. No changes in viability were observed in MB231 cells with all concentrations of VitC compared to control cells (Figure 1).

Therefore, for the subsequent experiments, we chose a dose that did not affect cell viability (0.1 mM) and one that reduced viability by 50% (10 mM) in MCF7. For comparison, we used the same doses to assess any molecular effects in MB231. 

**Figure 2:**
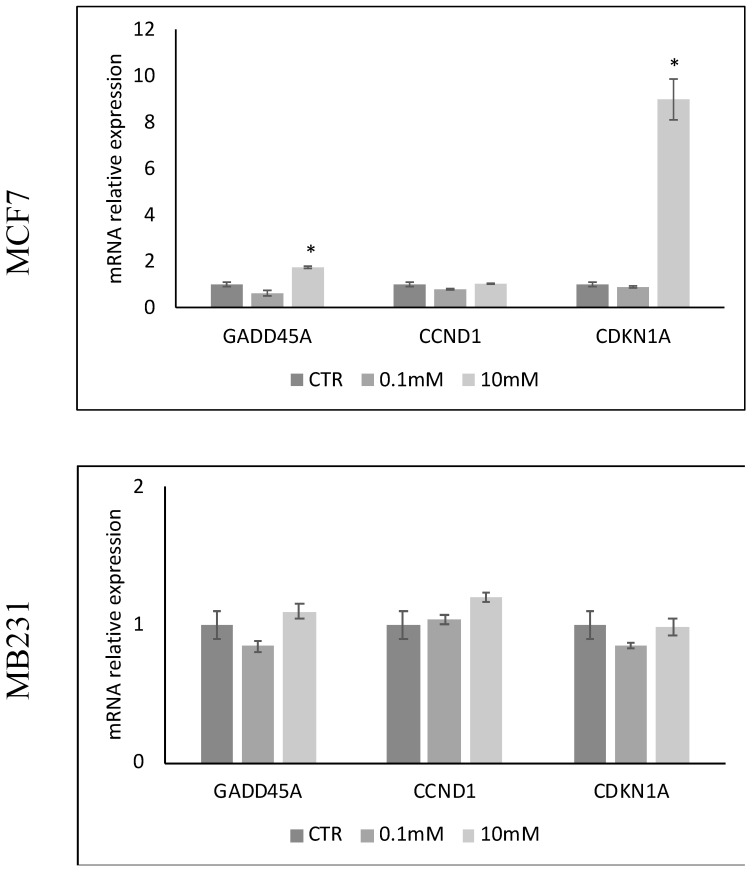
Effect of 0.1 mM and 10 mM Vitamin C on the gene expression of GADD45A coding growth arrest and DNA-damage-inducible alpha protein, CCND1 coding cyclin D1 protein, and CDKN1A gene coding cyclin-dependent kinase inhibitor 1A protein in MCF7 and MB231 cells. GAPDH and 18S rRNA were used as housekeeping genes. mRNA relative expression levels were calculated as 2^−ΔΔCt^, comparing the results of the treated samples with the control sample (CTR) equal to 1, the origin of the axes. Data are expressed as the mean ± SD of 3 independent experiments performed in duplicate. * *p* < 0.05 versus CTR.

We then sought to determine whether the low viability of MCF7 cells induced by high doses of VitC was accompanied by changes in the expression of genes involved in cell proliferation such as *CCND1*, *CDKN1a*, *GADD45A* (Figure 2). The results showed that 10 mM VitC was able to overexpress the gene for the cyclin-dependent kinase inhibitor (*CDKN1a*) and much more the gene for the protein responsible for growth arrest and DNA damage (*GADD45A*) only in MCF7 cells. The expression of *CCND1*, which encodes for cyclin D1 that stimulates the G1/S transition of the cell cycle, remained unchanged.

**Figure 3:**
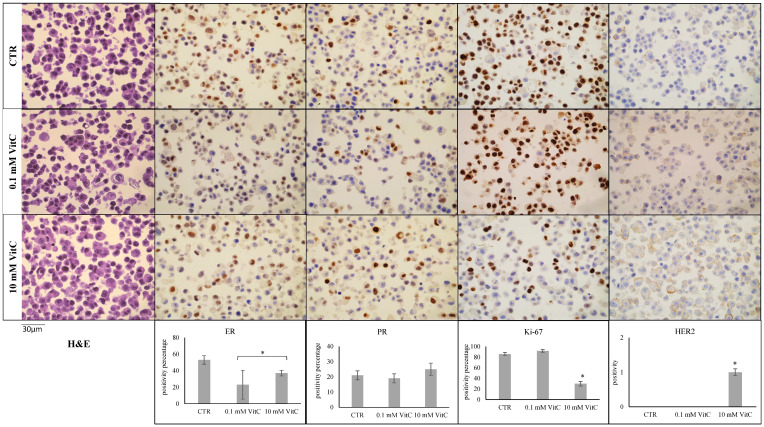
Effect of 0.1 mM and 10 mM Vitamin C on MCF7 cell phenotype. Immunocytochemistry analysis of ER, PR, Ki67, and HER2. Data represent the mean ± SD of ten slide readings from 2 experiments performed in duplicate. * *p* < 0.05 versus control sample (CTR).

Once the effects of 0.1 mM and 10 mM VitC on cell viability and cell cycle genes had been established, the phenotype of MCF7 and MB231 cells was analyzed using ER, PR, Ki67, and HER2 antibodies by immunocytochemistry. ER and PR positivity and HER2 negativity in MCF7 control cells and triple negativity in MB231 control cells were confirmed [26,28]. Twenty-four hours after treatment with VitC 0.1 mM and 10 mM, MCF7 cells showed a significant reduction in the level of ER expression (Figure 3). HER2 was upregulated with 10 mM VitC. The downregulation of Ki67 with 10 mM VitC was linked to the reduction of cell viability reported in Figure 1.

**Figure 4:**
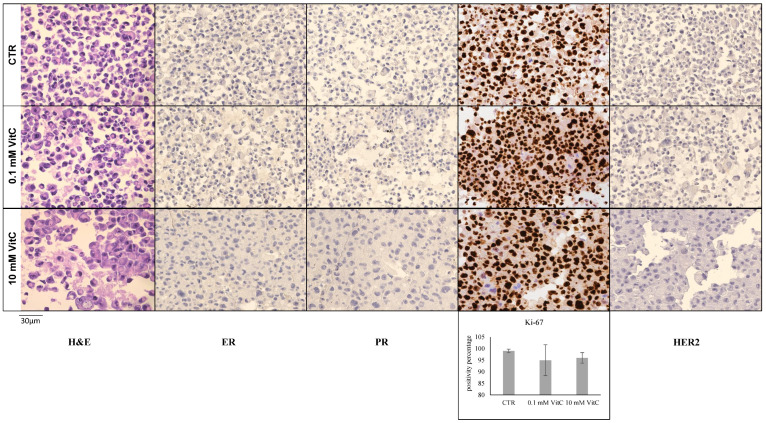
Effect of 0.1 mM and 10 mM Vitamin C on MB231 cell phenotype. Immunocytochemistry analysis of ER, PR, Ki67, and HER2. ER, PR, HER2 are negative. For Ki67, the data represent the mean ± SD of ten slide readings from 2 experiments performed in duplicate.

Furthermore, the concentration of 0.1 mM VitC did not influence the expression of Ki-67 and HER2. Interestingly, high-dose VitC reduced Ki-67 expression by 66% and induced HER2 expression. No effect was obtained with low and high concentrations of VitC in MB231 cells (Figure 4). There were no significant changes in the expression of the above-mentioned genes in MB231 cells. Overall, these experiments showed that treatment of MCF7 with a high dose of VitC results in a reduction of tumor cell growth. Moreover, VitC reduces the expression of ER and therefore it is possible that the cells do not respond to antiestrogen therapy as selective estrogen receptor degraders and modulators (tamoxifen) and aromatase inhibitors (anastrazole, letrozole, exemestane) [42].

### 2.2. Vitamin C Influences Sphingomyelin Metabolism in Human Breast Cancer 

Luminal A and triple-negative tumors are characterized by a different complexity of the lipid profile, including the Sph profile, which is much higher in the latter subtype [43]. Therefore, we sought to determine whether VitC caused different changes in Sph metabolism in MCF7 and MB231 cells. 

**Figure 5:**
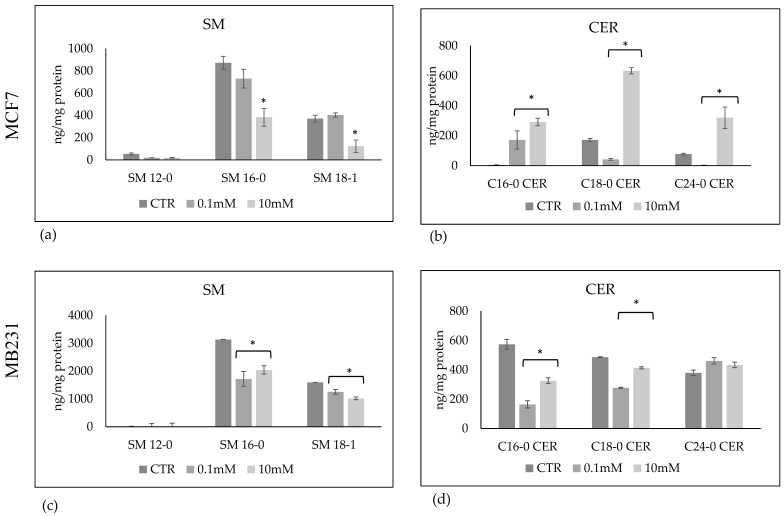
Sphingomyelin (SM) and ceramide (CER) species in MCF7 (**a**,**b**) and MB231 (**c**,**d**) cells treated with 0.1 mM and 10 mM VitC. Data are expressed as the mean ± SD of 3 independent experiments performed in duplicate. * *p* < 0.05 versus control sample (CTR).

To this end, we performed a lipidomic analysis in untreated and VitC 0.1 mM or 10 mM treated cells, using external calibrators 12:0 SM, 16:0 SM, 18:1 SM, 24:0 SM, 16:0 CER, 18:0 CER, 20:0 CER, 24:0 CER. The results show that 24:0 SM and 20:0 CER were absent in the samples. 0.1 mM VitC did not induce significant differences in SM species and increased the level of 16:0 Cer accompanied by a reduction of 18:0 CER and 24:0 CER in MCF7 cells. Interestingly, in the same cells 10 mM VitC significantly reduced 16:0 SM and 18:1 SM species (Figure 5a) and increased 16:0 CER, 18:0 CER and 24:0 CER (Figure 5b). In MB231 cells, both 0.1 mM VitC and 10 mM VitC reduced the SM and CER species (Figure 5c,d).

**Figure 6:**
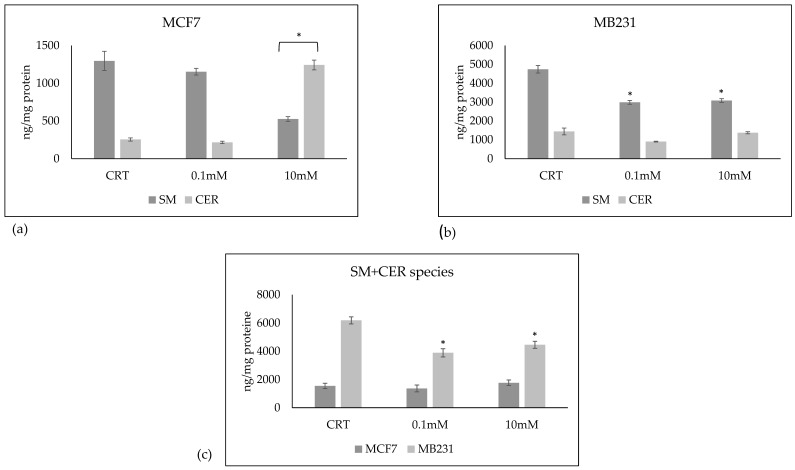
Total sphingomyelin (SM) and ceramide (CER) species in (**a**) MCF7 and (**b**) MB231 cells treated with 0.1 mM and 10 mM VitC. (**c**) Sum of SM and CER species in MCF7 and MB231 cells. Data are expressed as the mean ± SD of 3 independent experiments performed in duplicate. * *p* < 0.05 versus control sample (CTR).

Considering the total SM species analyzed, the value was 1297 + 127 ng/mg protein in MCF7 and 4744 + 204 ng/mg protein in MB231 (Figure 6a,b). The total value of the Cer species analyzed was 253 + 21 ng/mg protein in MCF7 and 1440 + 179 in MB231 (Figure 6a,b). These results confirmed that triple-negative tumor cells are richer in Sph content than luminal A cells. In fact, when adding SM and Cer, the total value is much higher in MB231 than in MCF7 (Figure 6c). VitC in MCF7 does not change the total value, justifying the reduction in SM and increase in Cer. In contrast, a reduction in the total value of Sph was observed in MB231, suggesting a possible reduction in the synthesis process (Figure 6c).

**Figure 7:**
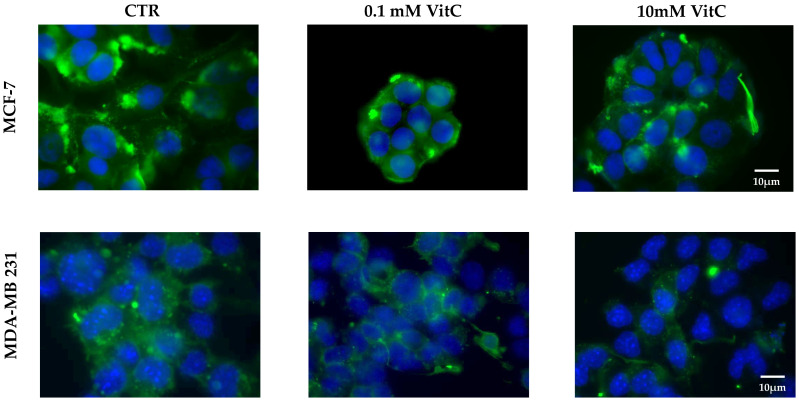
Presence of sphingomyelin in control (CTR), 0.1 mM, and 10 mM treated MCF7 and Mb231 cells with the EGFP-NT-Lys fluorescent probe.

The lower SM content in MCF7 cells compared to MB231 cells and its reduction after treatment with 10 mM VitC were confirmed by immunofluorescence using the EGFP-NT-Lys probe, as reported in the methods (Figure 7). It might be related to the reduced viability reported above. Future studies could clarify this point.

**Figure 8:**
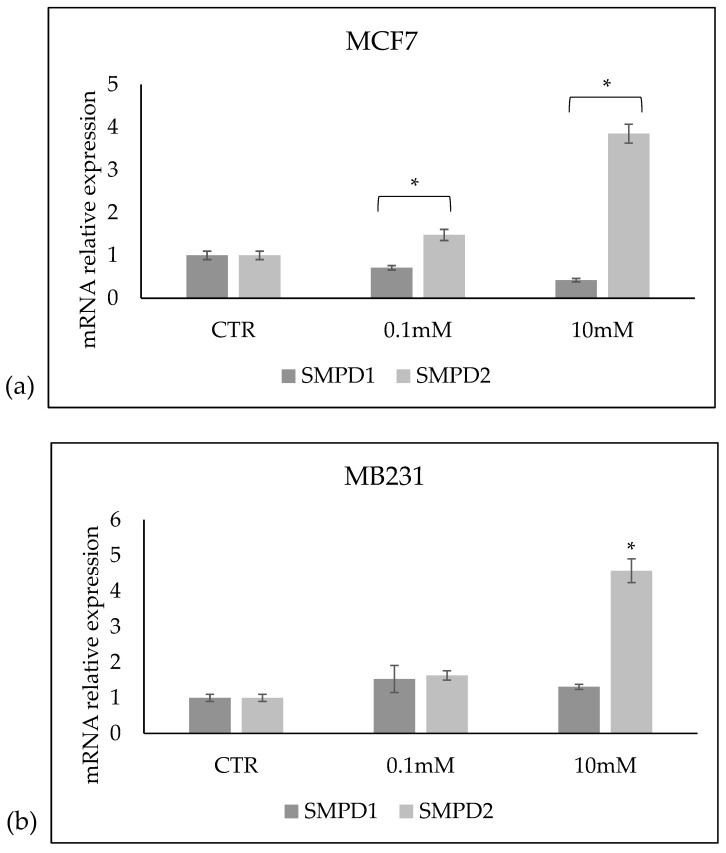
Effect of 0.1 mM and 10 mM Vitamin C on the gene expression of *SMPD1* coding for acid sphingomyelinase and *SMPD2* coding for neutral sphingomyelinase 1 in (**a**) MCF7 and (**b**) MB231 cells. GAPDH and 18S rRNA were used as housekeeping genes. mRNA relative expression levels were calculated as 2^−ΔΔCt^, comparing the results of the treated samples with the control sample (CTR) equal to 1, the origin of the axes. Data are expressed as the mean ± SD of 3 independent experiments performed in duplicate. * *p* < 0.05 versus CTR.

Once the effect of VitC on the Sph profile was identified, we sought to determine whether the changes were caused by the effect of VitC on the expression of aSMase or nSMase. As can be seen, 10 mM VitC reduced the expression of the *SMPD1* gene encoding for aSMase and overexpressed the *SMPD2* gene encoding for nSMAase1 in both cell lines (Figure 8). 

**Figure 9:**
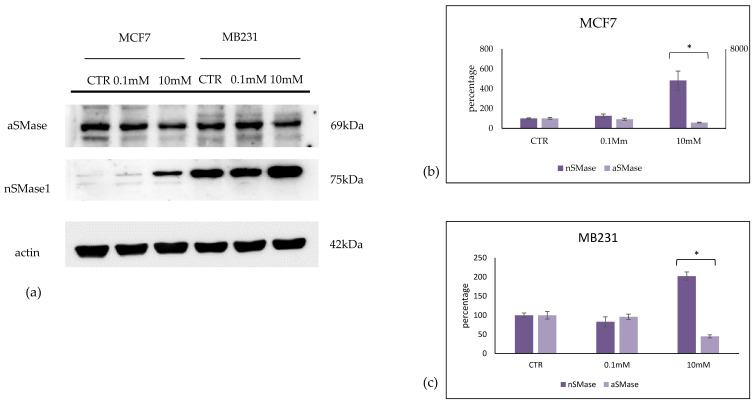
Effect of 0.1 mM and 10 mM Vitamin C on acid sphingomyelinase (aSMase) and neutral sphingomyelinase1 (nSMase) in MCF7 and MB231 cells. (**a**) Western blotting panel of aSMase and nSMase1 and actin as reference; (**b**) densitometric analysis of MCF7, the values were normalized with actin and were expressed as a percentage of the control sample (CTR). (**c**) densitometric analysis of MB231, the values were normalized with actin and were expressed as a percentage of the control sample (CTR). Data are expressed as the mean ± SD of 3 independent experiments performed in duplicate. * *p* < 0.05 versus CTR.

Consequently, 10 mM of VitC reduced acid sphingomyelinase (aSMase) and overexpressed neutral sphingomyelinase (nSMase) in both cell lines, although with truly higher values in MCF7 cells (Figure 9).

All together, these results indicate SM metabolism enzymes as possible targets of VitC.

## 3. Discussion

The overall results of this work suggest that high concentrations of VitC (10 mM) reduce the growth of the luminal A breast cancer cell line (MCF7) but not the triple-negative breast cancer cell line (MB231). 

The results do not agree with those of Lee et al. [44]. In fact, the authors obtained a reduction in cell viability of both MCF7 cells and MB231 cells. It is possible that the different result might be due to the culture and treatment conditions. In fact, the authors seeded the cells at a density double ours (2 × 10^4^ per well), did not change the medium before adding VitC and did not use antibiotics. Moreover, Mostafavi-Pour et al. used our own cell concentration and the same composition of the culture medium with antibiotics, but they waited for 70% confluence and did not change the medium before treatment with VitC [45]. Furthermore, the authors study non-pharmacological concentrations from 0.1 uM to 1 mM. In our case the 1 mM concentration had no effect in both cell lines. It is possible to hypothesize that the change in the medium that we made before the treatment with VitC was responsible for the different response of the cells. Furthermore, the company from which the cell lines were obtained was different from the one used by Lee et al. [44] and Mostafavi-Pour [45]. We verified the initial cellular phenotypes of the two cell lines with the marking of ER, PR, and HER2. This assured us that MCF7 was luminal A and MD231 was triple-negative. Therefore, the starting cells were in correct experimental conditions. Certainly, the different origins of cell lines and the different culture conditions compared to already published data [44,45] might influence the response of cells to high concentrations of VitC.

The results of this work shed light on a mechanism triggered by high doses of VitC on the luminal A tumor cell line that leads to a reduction in ER expression and the appearance of HER2 positivity. This result may be relevant for cancer progression even if VitC treatment led to a reduction in tumor burden. Indeed, the choice of hormonal treatment is based on histological type [46]. The administration of VitC at high concentrations could be the cause of an unexpected lack of response to classic hormone therapy [42]. The slowing down of tumor mass growth could be misleading and there could be a late but rapid deterioration due to resistance to hormonal treatment. In our very recent work, we described cholesterol metabolism and SM as growth media in luminal A breast cancer but not in triple-negative breast cancer [26]. Therefore, only luminal type A is (more easily) influenced by VitC and lipids. Here we report that high-dose VitC modulates the gene and protein expression of aSMase and nSMase by inhibiting the former and stimulating the latter in an almost balanced manner. Consequently, there is no SM degradation accompanied by an increase in Cer, indicating the permanence of a balance between the two molecules. Furthermore, both molecules are quantitatively reduced after VitC treatment, suggesting a possible reduction in synthesis rather than an activation of catabolism. It is known that Cer synthase is involved in cancer progression [47] and induces chemoresistance [48]. Therefore, the role of VitC in limiting the metastatic spread of triple-negative cancer cannot be excluded. Future studies may clarify this point. The effect of VitC on SM metabolism in Luminal A cells is different. There is a clear prevalence of increased nSMase1 over reduced aSMase, with a stimulation of SM catabolism and the production of high levels of Cer. At present, it is indeed difficult to determine whether increased Cer may be responsible for the reduction in ER expression and/or the appearance of HER2 positivity. A recent review reports that Sph metabolites, enzymes, and transport proteins are deregulated in human breast cancer cells and/or tissues and that Sph-driven mechanisms allow breast cancer cells to respond to or evade therapies [33]. This is the first study obtained in cell line in culture to show a simultaneous change in cellular phenotype and a change in SM metabolism with Cer production. It has been suggested that knowing the metabolism of Cer in luminal breast cancer may be a valuable aid for a new and correct therapeutic strategy [49]. In conclusion, the results are specific for luminal A breast cancer cell line. No changes in viability and molecular expression were observed in the triple-negative breast cancer cell line. The obtained results open the way for future studies aimed at demonstrating that high doses of VitC modify the Luminal A tumor phenotype via SM metabolism, i.e., by inhibiting nSMase.

Future research will be aimed at clarifying the metabolic mechanism that underlies the relationship between VitC and SM in MCF7, whether this occurs at the cell membrane level or whether there is an involvement of nuclear SM.

## 4. Materials and Methods

### 4.1. Chemicals

The cell lines MDA-MCF7 and MDA-MB-231 were produced by Elabscience Biotechnology (Houston, TX, USA). Cell culture medium (Dulbecco’s modified minimum essential medium, DMEM), fetal bovine serum (FBS), penicillin G, streptomycin, glutamine, and sodium pyruvate were produced by GIBCO Invitrogen (Carlsbad, CA, USA). Anti-neutral SMase (nSMase), anti-β-tubulin, anti-3-hydroxy-3-methylglutaryl-CoA reductase (HMGCR), and horseradish peroxidase-conjugated goat anti-rabbit secondary antibodies were produced by Abcam (Cambridge, UK). Ascorbic acid and 3-[4,5-dimethyl-2-thiazolyl]-2,5-diphenyl-2-tetrazoliumbromide (MTT) were produced by Sigma-Aldrich (St. Louis, MO, USA). The TaqMan SNP genotyping assay and reverse transcription kit were purchased from Applied Biosystems (Foster City, CA, USA). The RNAqueous^®^-4PCR kit was purchased from Ambion Inc. (Austin, TX, USA). SDS-PAGE molecular weight standards were purchased from Nzythech (Lisboa, Portugal). The chemiluminescence kit was purchased from Amersham (Rainham, Essex, UK).

### 4.2. Cell Culture and Treatments

MCF7 was used as luminal model A (ER+, PR+/−, HER2) and MDA-MB-231 as triple-negative model (ER−, PR−, HER2−) [21]. For the experiments cells at passages 5 and 6 were used. Cells were cultured in DMEM supplemented with 10% heat-inactivated FBS, 100 IU/mL penicillin/streptomycin and 200 mM L-glutamine. Cells were maintained at 37 °C in a saturating humidity atmosphere containing 95% air and 5% CO_2_. For the experimental model, VitC was added directly to the culture medium as shown below.

### 4.3. Cell Viability

The MTT assay was used to test cell viability, as previously reported [50]. MCF7 and MDA-MB-231 were seeded in 96-well plates at a density of 1 × 10^4^ cells/well with DMEM complete culture medium (see Section 4.2). After 24 h, the culture medium was removed and replaced with fresh complete DMEM medium (see Section 4.2). The same culture medium was used for control and experimental samples. Increasing concentrations of VitC from 0.1 mM to 20.0 mM were added and the cells were incubated for 48 h. As a control, samples with 1% or 2% DMSO were added due to their cytotoxic effect [51]. Then, MTT reagent was dissolved in 1x PBS and added to the culture at a final concentration of 0.5 mg/mL. After 3 h of incubation at 37 °C, the supernatant was carefully removed and the formazan salt crystals were dissolved in 200 µL of DMSO added to each well. Absorbance (OD) values were measured spectrophotometrically at 540 nm using an automated microplate reader (Eliza MAT 2000, DRG Instruments, GmbH, Marburg, Germany). Each experiment was performed twice in triplicate and the results were expressed as a percentage compared to the control cells.

### 4.4. Immunocytochemistry

The cell block was obtained by means of the Hologic protocol for the Cellient^®^Automated Cell Block System (Hologic, Marlborough, MA, USA) (Accessed on 28 April 2023) [https://www.hologic.com/sites/default/files/2020-07/MAN-06369-001_001_02.pdf]: in detail, the formalin-fixed cell cultures were first centrifuged at 1727 RPM for 10 min. After the removal of the supernatant, the cell precipitate was placed in a vial of Preservcyt^®^ solution (methanol-buffered solution) from the ThinPrep^®^ system (Hologic, Marlborough, MA, USA) and processed through Cellient^®^ instrumentation for 45 min, concentrating the cells and distributing them in a thin layer in a paraffin cell block. Subsequently, 4 µm sections were made and placed on positively charged slides, for immunocytochemical staining using the fully automated BOND III immunohistochemical stainer (Leica Biosystems, Nußloch, Germany): In detail, heat-induced antigen retrieval using BondTM Epitope Retrieval Solution 1 (Leica Biosystems, Newcastle Upon Tyne, UK, catalogue no. Catalogue No.: AR9961) on a ready-to-use citrate base for 30 min, an incubation of the primary antibody for 15 min with the ER (clone 6F11, RTU, Leica Biosystems, Newcastle Upon Tyne, UK, Catalogue No.: PA0151) and a heat-induced antigen retrieval with the ER. Catalogue No.: PA0151) and PR (clone 16, RTU, Leica Biosystems, Newcastle Upon Tyne, UK, Catalogue No.: PA0312) and for 30 min with Ki-67 (MM1) from Leica Biosystems, RTU, Newcastle Upon Tyne, UK, Catalogue No. Catalogue No.: PA0118; subsequently, the ready-to-use BondTM Polymer Refine detection system (Leica Biosystems, Newcastle Upon Tyne, UK, Catalogue No.: DS9800) was used to visualize the complex through a brown precipitate and hematoxylin for nuclear counterstaining. HER2 immunohistochemistry was performed using the HercepTest™ RTU (Glostrup, Denmark). For nuclear biomarkers (ER, PR and Ki-67), 10 different areas of approximately 0.4 μ2 each were selected using QuPath 0.2.0-m9 software (Accessed on 28 April 2023) (https://github.com/qupath/qupath/releases?page=3) in order to automatically count positive cells, the accuracy of which was then checked manually. Subsequently, the mean ± SD (standard deviation) of the percentage values obtained was used to quantify the positivity of the staining. Regarding the evaluation of HER2 staining, a score from 0 to 3+ was applied, according to the current ASCO-CAP guidelines [52].

### 4.5. Reverse Transcription Quantitative PCR (RTqPCR)

Total RNA was extracted from MCF7 cells cultured for 24 h, as reported above, using the RNAqueous-4PCR kit, as previously described [53]. Samples were treated with RNase-free DNase to prevent the amplification of any genomic DNA present. Samples were dissolved in RNase-free water and the amount of total RNA was quantified by measuring absorbance at 260 nm (A260). RNA purity was assessed by the A260/A280 ratio. The A260/A230 ratio was also used to indicate the presence of chemical contaminants in nucleic acids. The extracted RNA was immediately frozen and kept at −80 °C. Prior to cDNA synthesis, RNA integrity was assessed by 1.2% TAE agarose gel electrophoresis. The cDNA was synthetized using 1μg of total RNA for all samples using the High-Capacity cDNA Reverse Transcription kit under the following conditions: 50 °C for 2 min, 95 °C for 10 min, 95 °C for 15 s and 60 °C for 1 min, for a total of 40 cycles. The following target genes were analyzed: sphingomyelin phosphodiesterase 1 (*SMPD1*, Hs03679347^_^g1), sphingomyelin phosphodiesterase 2 (*SMPD2*, Hs55235), cyclin D1 (*CCND1*, HS00765553), Cyclin 1A-dependent kinase inhibitor (CDKN1A, Hs^_^00355782^_^m1), Growth Stop and DNA Damage Inducible Alpha (GADD45A, Hs_00169255_m1Glyceraldehyde-3-phosphate dehydrogenase (GAPDH, Hs_00187047^_^g1). (GAPDH, Hs999905^_^m1) and 18S rRNA (S18, Hs99999901^_^s1) were used as house-keeping genes. Relative mRNA expression levels were calculated as 2^−ΔΔCt^, comparing the results of treated samples with the control samples.

### 4.6. Protein Concentration and Western Blotting

The protein concentration was measured as previously reported with modifications [54]. Proteins (70 μg) were loaded into 10% SDS (sodium dodecyl sulphate) polyacrylamide gels by electrophoresis at 180 V for 60 min. The proteins were transferred onto a 0.45 μm cellulose nitrate strip membrane (Sartorius StedimBiotech S.A., Aubagne, France) in transfer buffer for 75 min at 100 V at 4 °C. The membranes were blocked for 60 min with 5% non-fat dry milk in PBS (pH 7.4) and incubated overnight at 4 °C with the specific antibodies for HMGCR, aS-Mase, or nSMase. The anti-βactin antibody was used to normalize the data. The blots were treated with HRP-conjugated secondary antibodies for 60 min. Band detection was performed with a balanced chemiluminescence kit from Amersham Pharmacia Biotech (Rainham, Essex, UK). Densitometric analysis was performed with Chemidoc Imagequant LAS500-Ge Healthcare-Life Science (Milan, Italy).

### 4.7. Ultrafast Liquid Chromatography–Tandem Mass Spectrometry

Lipid extraction and analysis were performed as previously reported [54]. The cell pellet was suspended in 10 mM Tris, pH 7.4, and diluted with 1 mL methanol. Three milliliters of ultrapure water and 3 mL of methyl tertiary butyl ether (MTBE) were added. Each sample was shaken for 1 min and centrifuged at 3000× *g* for 5 min. The supernatant was covered again. Extraction with MTBE was repeated on the pellet and the supernatant was added to the former. The organic phase was dried under nitrogen flow and resuspended in 500 μL of methanol. The molecules 12:0 SM, 16:0 SM, 18:1 SM, 24:0 SM, sphingosine-1-phosphate, sphinganine C18:0, C6:0 Cer, C8:0 Cer, C16:0 Cer, C18:0 Cer, C20:0 Cer, C24:0 Cer. C12:0 dihydroCer, arachidonoylglycerol (2AG), C16:0 glucosylceramide (GluCer), 16-0 18-1 phosphatidylcholine (PC), 16-0 20-4 PC, 18-1 18-0 standard PC were dissolved in chloroform/methanol (9:1 *v*/*v*) at a final concentration of 10 μg/mL. The stock solutions were stored at −20 °C. Working calibrators were prepared by diluting the stock solutions with methanol to final concentrations of 500:0, 250:0, 100:0, and 50:0 ng/mL. Twenty microliters of external standards or lipids extracted from serum with 12:0 SM and 6:0 Cer as internal standards (500 ng/mL) were injected after purification with specific nylon filters (0.2 μm). Analyses were performed using the ultra-performance liquid chromatography system with tandem mass spectrometer (Applied Biosystems, Monza, Italy). Lipid species were separated, identified, and analyzed as previously reported [45]. The liquid chromatography system was a Shimadzu Prominence UFLC (Milano, Italy), the pump was a Shimadzu LC-20 AD(Milano, Italy), the detector was an API 3200 linear triple quadrupole MS/MS(Milano, Italy), the injection valve was a Valco, the autosampler was a Shimadzu SR-20 AC HT(Milano, Italy), the column temperature stabilizer was a Shimadzu CTO-20A(Milano, Italy). The samples were separated on a Phenomenex Kinetex phenyl-hexyl 100 A column (50 × 4.60 mm diameter, particle diameter 2.6 μm) with a Phenomenex ULTRA phenyl-hexyl 4.6 pre-column safety protection. The column temperature was set to 50 °C. The column temperature was set to 50 °C and the flow rate to 0.9 mL/min. Solvent A was 1% formic acid; solvent B was 100% isopropanol containing 0.1% formic acid. The run was performed for 3 min in 50% solvent B and then in a gradient to reach 100% solvent B in 5 min. The system had to be reconditioned for 5 min in 50% solvent B before the next injection. Lipid species were identified using positive turbo-ion spray (ESI) and multiple reaction mode monitoring. The voltage of the ion spray was 5.4 kV, gas 1 was air, gas 2 was nitrogen, the temperature was 650 °C and the flow rate of the curtain gas 40.5 L/h. The collision gas flow was maintained at 5.0 L/h. Data were acquired and processed using AnalystTM and Analyst 1.5 software on a Dell Precision T3400 personal computer with a Samsung ML-2851 MD graphics printer (Milano, Italy).

### 4.8. Immunofluorescence

Immunofluorescence of SM was performed as reported according to Albi et al. [55]. The SM localization was studied by using the SM probe, enhanced green fluorescent protein-nontoxiclysenin (EGFP-NT-Lys). The probe was purified from E. Coli strain BL21 (DE3) harboring pET28/EGFP-NT-Lys according to Tomishige et al. [56], with a little modification. In brief, after the bacteria culture reaches OD600 = approx. 1, the expression of EGFP-NT-Lys was induced at 18 °C for two overnights in the presence of 125 µM IPTG (VWR life science, Radnor, PA, USA). EGFP-NT-Lys was purified by Hitrap TALON crude column (Cytiva, Munzinger Str. 5, 79,111 Freiburg im Breisgau, Germany) from bacteria lysate using its His-tag. Imidazole used in the elution of the protein from the column was removed by dialysis. The dialyzed protein was mixed with glycerol (VWR chemicals, Radnor, PA, USA) at 50% (*v*/*v*) and stored at −20 °C. On the day of the experiment, the medium was removed, cells were washed with DMEM/5% lipid depleted serum (LPDS) and treated with 15 µg/mL EGFP-NT-Lys diluted in DMEM/5% LPDS for 45 min. Then, the cells were fixed with 250 µL of 4% paraformaldehyde (PFA) at room temperature (RT) for 30 min. After washing, the residual PFA was neutralized by 0.1 M NH4Cl at RT for 15 min. The cells were washed three times with 500 µL of PBS and nuclei counterstained with DAPI. Coverslips were mounted and cells viewed in a DMRB Leica epi microscope equipped with a digital camera.

### 4.9. Statistical Analysis

Three independent experiments performed in duplicate were carried out for each analysis. Data are expressed as mean ± SD; Student’s *t*-test was used for statistical analysis. To compare the effect of 0.1 mM VitC versus CTR and/or 10 mMVitC, ANOVA test was used.

## 5. Conclusions

In summary, we have shown how the administration of high doses of VitC on Luminal A breast cancer cells is responsible for the reduction of tumor growth but also for their phenotypic change towards cells resistant to hormone therapy with a possible activation of the catabolism of SM and production of high levels of Cer.

## Data Availability

Data are contained within the article.
